# Scale-up evaluation results of a multi-level approach using mailed immunochemical test (FIT) and patient navigation to increase colorectal cancer screening rates in rural clinic settings

**DOI:** 10.1186/s12875-026-03428-w

**Published:** 2026-07-14

**Authors:** Jessica J. Currier, Jennifer Coury, Emily Myers, Erin S. Kenzie, Amanda F. Petrik, Brittany Badicke, Margaret McLain McDonnell, Maryan Carbuccia Abbott, Gloria D. Coronado, Melinda M. Davis

**Affiliations:** 1 Knight Cancer Institute, Oregon Health & Science University, 3181 SW Sam Jackson Park Rd., Portland, OR 97239 United States of America; 2 Oregon Rural Practice-based Research Network, Oregon Health & Science University, 3181 SW Sam Jackson Park Rd., Portland, OR 97239 United States of America; 3 Kaiser Permanente Center for Health Research, 3800 N. Interstate Avenue, Portland, OR 97227 United States of America; 4 Department of Family Medicine, Oregon Health & Science University, 3181 SW Sam Jackson Park Rd., Portland, OR 97239 United States of America; 5 OHSU-PSU School of Public Health, Oregon Health & Science University, 1810 SW 5th Avenue, Portland, OR 97201 United States of America; 6 Cancer Center, University of Arizona, 1200 E University Blvd, Tucson, AZ 85721 United States of America

**Keywords:** Colorectal cancer screening, Mailed fecal immunochemical testing, Patient navigation, Scaling-up interventions, Implementation science, Rural health, Multilevel intervention

## Abstract

**Background:**

Colorectal cancer (CRC) screening effectively reduces CRC mortality and morbidity, yet screening rates remain below US national targets in rural and low-resourcedd settings. An effective intervention to improve screening is mailed fecal immunochemical tests (FIT) combined with patient navigation. However, these multicomponent interventions have been unevenly adopted across health systems and organizations, and studies of how to spread effective system-level approaches are limited.

**Methods:**

We conducted a mixed-methods evaluation of a multi-component scale-up approach designed to increase CRC screening rates in rural, low-resourced primary care clinics, based on findings from the SMARTER CRC pragmatic trial. The approach included five scale-up activities: (1) an Extension for Community Healthcare Outcomes (ECHO) tele-mentoring program; (2) patient navigation training; (3) webinars on process design and communication strategies; (4) technical assistance; and (5) dissemination of the *Colorectal Cancer Screening Outreach for Rural Populations Facilitation Guide*. We measured engagement in scale-up activities, adoption of mailed FIT and patient navigation outreach, and modifications to organizations’ CRC screening programs.

**Results:**

A total of 47 participants from 25 organizations participated in scale-up activities. Participants were most commonly from clinical practices (28%), hospital-based systems (24%), and community organizations (20%). All organizations participated in the ECHO series (100%, *n* = 25), and some also engaged in additional scale-up activities. Most organizations (84%, *n* = 21) reported having a CRC screening program at baseline. Engagement in ECHO and other scale-up activities influenced adoption and modification of CRC screening patient outreach strategies for most organizations (69%, *n* = 17). Participation led to modifications in existing programs.

**Conclusion:**

Participation in multifaceted scale-up activities increased organizational knowledge and engagement in CRC screening patient outreach within rural, low-resourced clinic settings. These activities also supported the refinement of existing CRC screening programs through the adoption and modification of outreach strategies. These findings can inform future efforts to optimize scale-up approaches for CRC screening and other complex interventions in rural, low-resourced healthcare settings.

**Trial registration:**

Registered at clinicaltrial.gov (NCT04890054) and at the NCI’s Clinical Trials Reporting Program (CTRP # NCI202101032) on May 11, 2021.

**Supplementary Information:**

The online version contains supplementary material available at https://doi.org/10.1186/s12875-026-03428-w.

**Table:** 

Text box 1. Contributions to the literature
• The research describes a mixed-methods evaluation of a multicomponent scale-up intervention comprised of five components supporting adoption of the population outreach activities (e.g., mailed FIT and patient navigation) tested in the SMARTER CRC pragmatic trial in rural healthcare organizations.
• We describe how organizations were engaged in multifaceted scale-up activities and how their engagement influenced adoption and modification of CRC screening patient outreach strategies, specifically mailed FIT and navigating patients with an abnormal FIT result to follow-up colonoscopies.
• Findings may inform future approaches to optimize scale up for CRC screening and other complex interventions in rural, low resourced health care settings.

## Introduction

Colorectal cancer (CRC) is the third most common cause of cancer-related death among men and women in the United States [1–3]. CRC is 91% curable with timely detection and appropriate treatment of precancerous growths [4]. CRC screening is a highly effective way to reduce CRC mortality and morbidity, yet screening rates remain persistently lower than US national targets [5–7]. Prior research estimates that 28 million US adults aged 45–75 (33%) have never been screened for CRC [8].

Systematic approaches to screening outreach can help increase rates of screening and have been shown to improve health outcomes. For individuals with an average risk of CRC, mailing a fecal immunochemical test (FIT) is an evidence-based approach to raising rates of cancer screening in a given population [9]. Mailed FIT has demonstrated average screening improvements of 22–28% [5, 10–13]. It is an appropriate first step in CRC screening for individuals with no family history of CRC; abnormal FIT results indicate when people need further surveillance with a colonoscopy. Another effective approach to improving screening over usual care is patient navigation to colonoscopy, which can support individuals with abnormal FIT test results to complete a follow-up colonoscopy [14, 15]. A multi-component outreach of mailed FIT combined with navigating patients to colonoscopy can be particularly helpful in closing the gaps in screening rates between different populations [15–17]. Despite effectiveness, patient outreach activities involving mailed FIT and patient navigation have been unevenly adopted by health systems and organizations due to multi-factorial implementation challenges [18, 19]. 

To improve implementation of these proven effective outreach strategies, we conducted a cluster-randomized pragmatic trial, Screening More patients for CRC through Adapting and Refining Targeted Evidence-based Interventions in Rural settings (SMARTER CRC). SMARTER CRC was a collaboration between Medicaid health plans and rural primary care clinics to support implementation of a mailed FIT and patient navigation program that consisted of three phases: a pilot, implementation study, and scale-up. Materials were tailored for rural settings during the pilot phase from December, 2019 – September, 2020 and tested in a pragmatic trial during the implementation [20]. Results of the pragmatic trial, conducted between May 2020 and February 2021, demonstrated effectiveness among the 28 participating rural primary care practices in rural Oregon; eligible rural, Medicaid beneficiaries in intervention clinics were 7.5% (CI: 95%) more likely to be screened for CRC than those in the control clinics [21]. 

The scale-up phase aimed to expand effective SMARTER CRC program components (e.g., mailed FIT and patient navigation) to additional settings serving rural patient populations [18, 19]. Insights from the SMARTER CRC pragmatic trial directly informed the scale-up phase by identifying implementation barriers, adaptation needs, and dissemination strategies needed to support broader adoption. In this manuscript, we report results from our evaluation of the scale-up phase of the SMARTER CRC study. Our key research questions were to examine: (1) how organizations engaged with a multi-component scale-up intervention; (2) which CRC screening patient outreach activities (e.g., mailed FIT and patient navigation outreach) were adopted through participation in scale-up activities and why; and (3) what modifications organizations made to their existing CRC screening program as a result of participation in this scale-up. The objective of the evaluation was to characterize participating organizations with respect to: (1) their engagement in the ECHO and other scale-up activities; (2) adoption of the SMARTER CRC program components (i.e., mailed FIT and patient navigation outreach); (3) and modifications made to the program components.

## Methods

SMARTER CRC obtained approval from the Oregon Health and Science University Institutional Review Board (protocol number: 20681), which granted a waiver of informed consent. A ceding agreement was obtained from the Kaiser Permanente Center for Health Research.

### Recruitment of participating organizations

We recruited clinical practices, hospital systems, community organizations, health departments, health plans, and tribal health systems operating in low-resourced rural settings in Oregon [18]. Organizations were invited to participate based on their interest in CRC screening and organizational change. To be eligible for participation, organizations needed to demonstrate interest in improving rates of CRC screening and/or follow-up in their patient populations, commit to attending the ECHO sessions, and completing data collection activities. Our method of choosing organizations involved developing a list of potential organizations for scale-up outreach that included organizations unable to take part in the initial pragmatic trial but interested in the proposed research, contacts of advisory board members and their networks, organizations with previous collaborators of study team members, and those submitting ECHO registration requests. Additional leads were identified through snowball sampling, warm handoff referrals, and direct inquiries who learned about the project through regional or national presentations. Study participants were staff members employed by organizations enrolled in this study. Recruitment of organizations enrolled in this study occurred between July 2022 and September 2023. 

### Scale-up intervention

The scale-up intervention was comprised of multiple activities designed to spread the SMARTER CRC program and increase CRC screening rates across participating rural healthcare organizations [19]. Findings from the cluster-randomized trial SMARTER CRC of a mailed FIT and patient navigation program were used to inform and tailor the multicomponent scale-up activities [18, 19, 22, 23]. Scale-up activities consisted of collaborative learning, a facilitation guide titled Colorectal Cancer Screening Outreach for Rural Populations, individualized technical assistance, a patient navigation workshop, and webinars (Table 1) [18]. Two Extension for Community Healthcare Outcomes (ECHO) tele-mentoring courses comprising of 6-sessions were delivered in fall 2023 (09/14/23 − 11/30/23) and spring 2024 (03/23/24 − 06/01/24). ECHO session topics are shown in Table 1.

**Table 1:** Scale-up activities, description, measures & data sources

Scale-up Activity	Description	Measures	Evaluation Data Sources
ECHO	A tele-mentoring program focused on primary care quality improvement that connects healthcare professionals with subject matter experts through a series of didactic and peer-to-peer learning sessions. *ECHO series topics*: 1. Building the business case for CRC outreach 2. Building engagement, (3) designing a mailed FIT program 3. Identifying patients who are due for CRC screening 4. Delivering patient communications (FIT and reminders) 5. Navigating to follow-up colonoscopy	• Organization and staff level of participation • Characteristics of participating organizations and staff • Organization and staff satisfaction with ECHO	• Registration & attendance • ECHO Session Observation Notes • Pre-ECHO survey • Post-ECHO survey • 6-mos post-ECHO survey • Key-informant Interviews
Patient Navigation Workshop	5-hour workshop addressed the importance of CRC screening and follow-up, barriers to CRC screening, and the patient navigation protocol	Organization and staff level of participation; participant satisfaction with patient navigation workshop	Registration & attendance; Post Patient Navigation Workshop Survey; Key-informant Interviews
One-on-one technical assistance	One-on-one support addressing an organization’s goals & objectives	Level of participation; participant satisfaction with technical assistance	Technical Assistance Meeting Notes; Key-informant Interviews
Training Webinars	1-hour webinar series facilitating specialty training in CRC screening patient outreach & best practices	Level of participation; participant satisfaction with training webinars	Post-Webinar Survey; Key-informant Interviews
Colorectal Cancer Screening Outreach for Rural Populations Facilitation Guide	Information & resources supporting continuing education on mailed FIT and patient navigation	Level of participation; participant satisfaction with facilitation guide content	Key-informant Interviews

Each ECHO course (hereafter referred to as a cohort) was facilitated by academic researchers and industry professionals with expertise in population-based CRC screening and included didactic presentations from subject experts in CRC screening. All other scale-up activities were led by CRC screening subject matter experts who were also members of the research team. The research team was available by phone or email to provide individualized support to staff from participating organizations.

### Evaluation method

We conducted a mixed-methods evaluation of a multi-component scale-up approach designed to increase CRC screening rates in rural, low-resourced primary care clinics, based on findings from the SMARTER CRC pragmatic trial. Our approach was guided by Reach, Effectiveness, Adoption, Implementation, Maintenance (RE-AIM) [24] and other implementation science frameworks, including Clinic Sustainability Assessment Tool (CSAT) [25], Program Sustainability Assessment Tool (PSAT) [26], Framework for Reporting Adaptations and Modifications to Evidence (FRAME) [27], Framework for Reporting Adaptations, Modifications to Evidence-based Implementation strategies (FRAME-IS) [28], and Going to Full Scale [29] to assess engagement, adoption, implementation, maintenance, and modification of CRC patient outreach activities by program component (mailed FIT and patient navigation). We drew upon multiple implementation science frameworks to provide a comprehensive understanding of different factors that influenced organizational engagement scale-up activities and adoption and modification of CRC screening patient outreach by program component (Mailed FIT and patient navigation), including contextual factors that enable or prohibit sustainable clinic processes and workflows, and sustainability of a CRC screening patient outreach program.

**Fig. 1:**
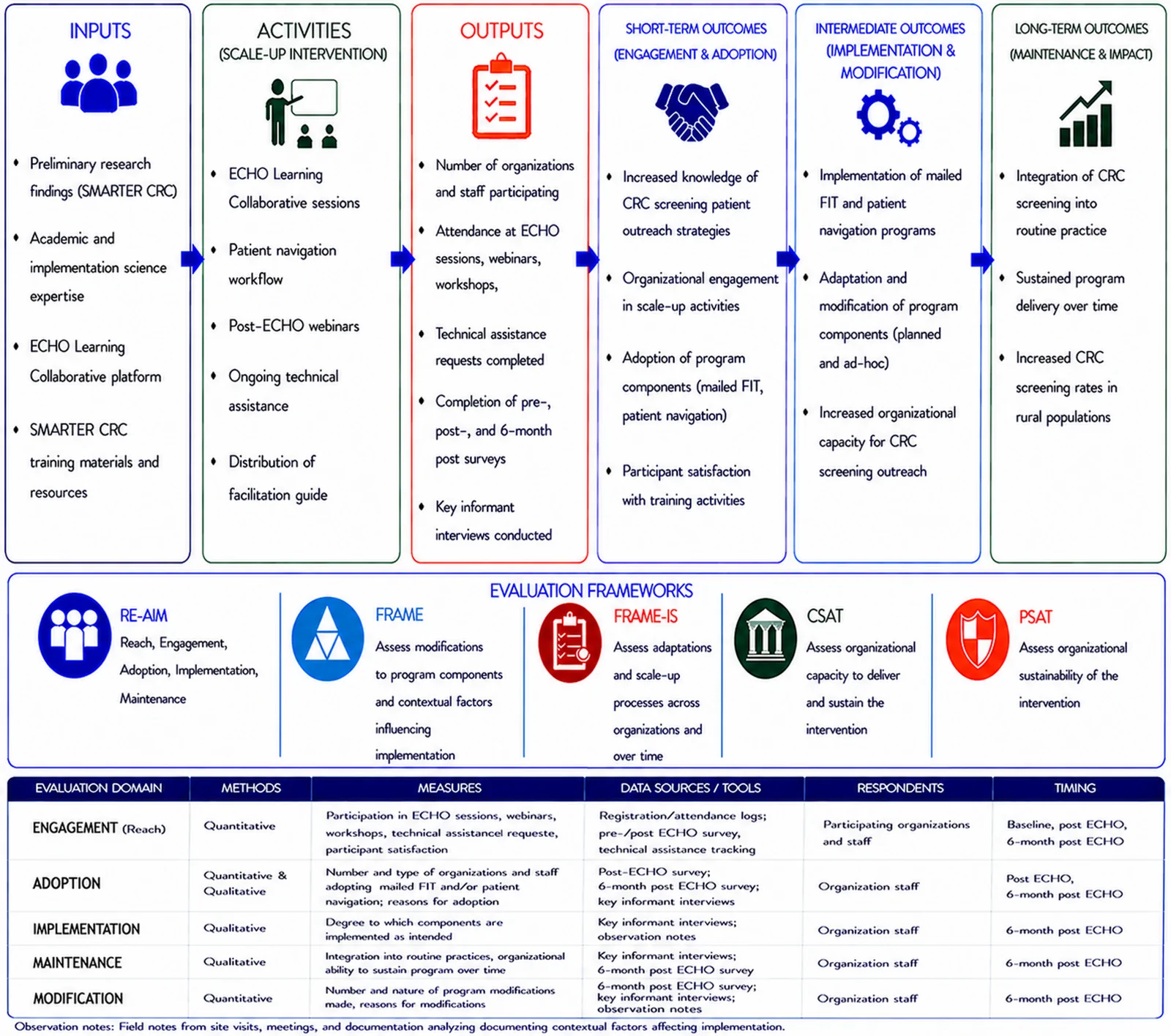
Logic model scale-up evaluation measures, data sources, and implementation frameworks

The evaluation focused on three key outcome variables that include engagement in scale-up activities, adoption of CRC screening patient outreach program components and modifications to CRC patient outreach components. We defined engagement as sustained participation of healthcare organizations in mechanisms that instigate and sustain a specific healthcare improvement activity [30]. Engagement in scale-up activities was measured using organization and staff level of participation (i.e., participant registration and attendance) and satisfaction with scale-up activities. We defined adoption in alignment with the RE-AIM framework [24], where adoption is the absolute number, proportion, and representativeness of settings and intervention agents (people who deliver the program) who are willing to initiate a program and why [24, 31]. Adoption was assessed by the number and type of organizations implementing core patient outreach program components (mailed FIT and patient navigation), using post-ECHO and 6-month surveys. Reasons for adoption and the degree to which an organization implemented CRC outreach activities by program component were explored through key-informant interviews with staff from participating organizations. Implementation (extent of program delivery) and maintenance (integration into routine practice) was assessed qualitatively via interviews. We also documented modifications that organizations made to existing CRC screening programs (i.e. mailed FIT and/or patient navigation) [28]. In this paper, we use the term “modification” broadly to describe changes to an intervention that occur in an ad hoc or unplanned manner [32]. Program modifications, including their number, type, and whether they were ad hoc or planned, were assessed using surveys and interviews. Interviews also explore reasons for modifications. We also evaluated the rationale for modification, and fidelity consistency with the CRC screening program evidence-based intervention, (i.e. mailed FIT and patient navigation) [18, 19, 27]. Evaluation measures, data sources, and application of implementation frameworks are shown in Fig. 1.

### Data collection tools and methods

We collected data from multiple sources and at three time points: (1) at baseline prior to an organization’s participation in scale-up activities (i.e., baseline); (2) immediately following the ECHO training (i.e., post-ECHO); and (3) 6-months after the ECHO (i.e., 6-months post-ECHO). To understand CRC screening program adoption, we assessed each participating organization’s CRC patient outreach activities at these timepoints.

Surveys were administered to between 1 and 4 staff members from participating organizations at each of these timepoints. All surveys were disseminated electronically to the same staff member to document change over time. The baseline survey was informed by the RE-AIM implementation framework [24]. The post-ECHO survey was disseminated electronically immediately after the final session of the 6-part ECHO series. The survey included questions about attendance, satisfaction with the ECHO content and format, level of participation in the ECHO, adoption, adoption facilitators, and modifications of SMARTER CRC program components (i.e., evidence-based patient outreach). Content from the CSAT and PSAT was included in the post-ECHO survey. The 6-month post ECHO survey was disseminated 6 months after the ECHO series concluded. The survey consisted of several questions assessing adoption, adoption facilitators, modifications, and sustainability. The survey was informed by the CSAT, PSAT, FRAME, and FRAME-IS, and Going to Full Scale frameworks to measure modifications made to a CRC patient outreach program [24, 26, 28, 31, 33]. 

In addition to measuring our primary outcomes (e.g., engagement, adoption, modification) using the baseline, post-ECHO, and 6-month post-ECHO surveys, we also used registration and attendance data for each scale-up activity, and observation notes documenting ECHO participant interactions to measure engagement. Semi-structured interviews with staff from participating organizations assessed program adoption and modifications.

The semi-structured interview guide was informed by the RE-AIM, CSAT, and PSAT implementation science frameworks. Interview questions explored participants’ engagement with the ECHO and other scale-up activities, as well as subsequent changes to mailed FIT and patient navigation patient outreach activities. Interviewees were asked to describe their ideal CRC screening patient outreach program to assess motivation for participating in one or more scale-up activity, and to potentially draw connections to the adoption of mailed FIT or patient navigation. Key informant interview participants were identified using ECHO session attendance (> 80% attendance) and holding a leadership position within their respective organizations (*n* = 26). All interviews were conducted virtually via Zoom between August 2023 and March 2024. Audio recordings were professionally transcribed via Rev.com, transcripts were reviewed and validated for accuracy, and final transcripts were uploaded into Atlas.ti (version 15) for coding and thematic analysis.

### Data analysis

Survey data was exported from REDCap and cleaned prior to analysis. Surveys with a minimum of 70% required responses completed were kept for analysis. Given the assumption of a normal distribution of participants, a parametric analysis was used. Demographic statistics (participant’s county, race and ethnicity, healthcare role, rurality) were analyzed and reported for all registrants that participated in at least one session. Descriptive statistics are presented for all variables reported.

For the analysis, a two-tier organizational analysis and an assessment of participant survey responses was performed. For the organization analysis, participants were clustered and compared based on their respective organizations, and the differences between the organizational providers assessed. Participants were also compared based on their individual roles within their organizations. For the survey analysis, responses were assessed for satisfaction with the ECHO trainings and the adoption, scale-up, and impact of the trainings in the respondents’ organizational screening practices. All quantitative data management and statistical analyses were performed using Stata/BE version 17.0 (StataCorp, College Station, TX, USA).

Qualitative analysis of interview transcripts was guided by the thematic analysis approach described by Braun and Clarke (2008) including data familiarization, code development, and theme identification [34]. Two qualitative analysts (JC and EM) developed a codebook using an inductive, open coding approach informed by the interview guide and survey questions. Codes were applied to meaningful segments of text to capture key concepts and patterns in the data. Codes were then organized into themes and sub themes to identify patterns and relationships, with further grouping into higher-level thematic categories. Themes were reviewed, defined, refined and documented through a thematic memo. Although the approach was informed by Braun and Clarke’s framework [34], we did not develop a formal conceptual model. Principles of grounded theory informed the analysis by supporting an inductive process of coding, categorization, and iterative refinement of themes to ensure that interpretations were grounded in the data.

## Results

### Participant characteristics (Reach)

A total of 25 organizations participated in scale-up activities, represented by 47 individual staff members who engaged in activities and completed study measures. Characteristics of participating organizations and their staff representatives are shown in Table 2. In addition, 11 key-informant interviews were conducted with staff representatives from participating organizations enrolled in the study (8 from the spring cohort and 3 from the fall cohort).

**Table 2:** Organization and staff characteristics

Organizations	*N* (%)
**Organization Type**	(***N* =** )
Clinic or practice	7 (28%)
Hospital-based system	6 (24%)
Community organization	5 (20%)
Health plan/accountable care organization	2 (8%)
Health system	2 (8%)
Tribal health system or health center	2 (8%)
State health department	1 (4%)
**Staff Role**	(***N*** **=** **47**)
Health outreach coordinator	16 (34%)
Nursing staff	10 (21%)
Administrative staff	9 (19%)
Clinician	6 (13%)
Practice management	5 (11%)
State health department staff	1 (4%)

At baseline, 84% of organizations (*n* = 21) reported having an existing CRC screening program. Of those organizations, 57% (*n* = 14) reported that the screening program included mailed fecal immunochemical tests (mailed FIT), and 43% (*n* = 11) of those organizations’ CRC screening programs included patient navigation to follow-up colonoscopy after abnormal mailed FIT results. One organization (4%) implemented a mailed FIT program after participating in the ECHO. Two organizations (8%) reported plans to implement CRC screening patient outreach activities after participating in the ECHO, training webinars, and the patient navigation workshop.

### Engagement with scale-up activities (Reach)

Across both ECHO cohorts (i.e., spring and fall), average attendance per session was 31 participants (range: 23 to 39), with 84% attending at least one ECHO session (Table 3). Participants attended an average of 4 of 6 sessions. Attendance was higher in the spring ECHO cohort (14 organizations; mean 19 participants/session; range: 14 to 22 participants/session), compared to the fall cohort (11 organizations; mean 13 participants/session; range: 7 to 17 participants/session). Staff with participating organizations described the ECHO as a worthwhile experience. Participants described the ECHO as a convenient and positive learning environment. The virtual format was a convenient format, appropriate time commitment, fostered peer-to-peer learning, and included relevant CRC screening patient outreach strategies and resources.

**Table 3:** Scale-up activity offerings and attendance

Post-ECHO Scale-up Activity	Number of Offerings *n* = 8	Number of Organizations *n* = 25	Number of Participants *n* = 47
ECHO spring cohort	1	14 (56%)	19 (40%)
ECHO fall cohort	1	11 (44%)	13 (28%)
Patient Navigation workshop	2	5 (20%)	13 (28%)
Patient engagement webinar	1	7 (28%)	10 (21%)
Outreach to diverse populations webinar	1	Data Unavailable	20 (43%)
Technical assistance meetings	2	2 (8%)	2 (4%)

Organizational engagement in all other scale-up activities was lower, ≤56% (Table 3). The two patient navigation workshops were attended by 20% of organizations (*n* = 5; 28% of participants; *n* = 13 participants). Two training webinars were offered. The Patient Engagement webinar was attended by 28% of organizations (*n* = 7; 21% of participants; *n* = 7 participants). While data on the number of organizations attending the Outreach to Diverse Populations webinar is unavailable, the webinar was attended by 43% of participants (*n* = 20). Average webinar attendance was 15 (32%) participants. One-on-one technical assistance was provided upon request. Two organizations (8%) requested technical assistance with members of the research team. 

## Adoption of CRC screening patient outreach strategies (Adoption) 

### Adoption intentions and influence of ECHO

The majority of organizations (79%, *n* = 20) reported plans to use knowledge gained through ECHO to develop, modify, or improve CRC screening patient outreach activities (e.g., mailed FIT and patient navigation). Participants reported in the surveys and interviews that the ECHO influenced the organizations’ current and future CRC screening patient outreach activities. By enhancing awareness and providing essential skills and resources, the ECHO initiative supported participating organizations’ CRC screening outreach efforts and served as a catalyst for program expansion and quality improvement. One participant stated, “I think it (ECHO) confirmed what we are already doing and assisted with some obstacles others were facing to help us learn workarounds.” (Participant ID 125).

### Adoption of evidence-based intervention components

**Fig. 2:**
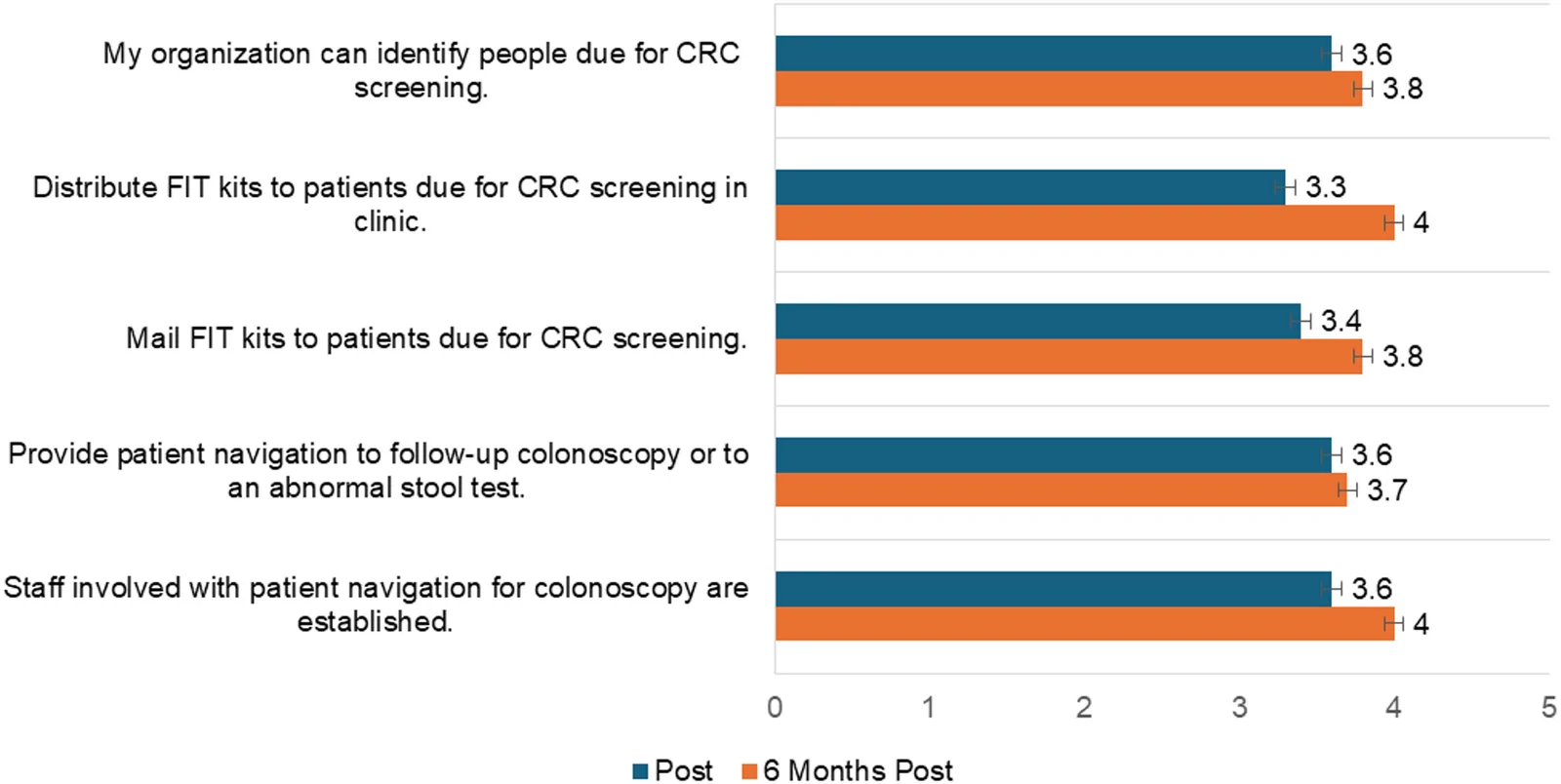
CRC ECHO influence on provider practice activities. Post ECHO session and 6 months post ECHO session, (*N* = 20)

Most organizations (69%, *n* = 17) reported that participation in the ECHO influenced their current CRC screening practices, and 63% (*n* = 16) reported plans to implement practice changes. Participants strongly agreed that practice activities relating to SMARTER CRC program components changed as a result of their ECHO participation, and this agreement was even greater at 6-months after the ECHO series (Fig. 2).

Adoption activities occurred at both the organization and patient levels. Organization-level changes included hiring of bilingual staff to support their Spanish-speaking patients; enhancing FIT kits with visual aids and a pen; offering transportation to colonoscopy; and guiding patients through colonoscopy screening. Efforts to raise awareness of CRC screening and FIT testing involved strategies such as informational displays in clinic waiting areas, sending birthday-based screening reminders, and hosting mobile medical van CRC screening events. Some organizations implemented patient incentives for completed CRC screenings, offering small items such as a poop emoji pen, toilet paper, or gift cards to local grocery stores or gas stations. Patient-level strategies focused on reducing screening barriers, tailoring outreach messaging, increasing knowledge and awareness of CRC screening, and encouraging participation through targeted incentives.

The primary motivation for adoption was increasing CRC screening rates for early detection. Specific activities included hiring dedicated staff to lead CRC screening outreach activities, offering CRC screening training and education to clinic staff, and workflow redesign. Organizations shared that they increased staff and provider awareness of new CRC screening outreach activities and workflow processes during clinic and leadership meetings, annual retreats, and “lunch and learn” sessions. Adoption of new workflow processes consisted of procedures for in-clinic FIT distribution, tracking the return of FIT kits mailed to patients, assigning lab orders to FIT kits, and developing phone, email, and text scripts for staff to use for pre-visit planning with patients, and documenting follow-up procedures for patients with an abnormal FIT result.

### Facilitators and barriers to adoption (implementation context)

**Fig. 3:**
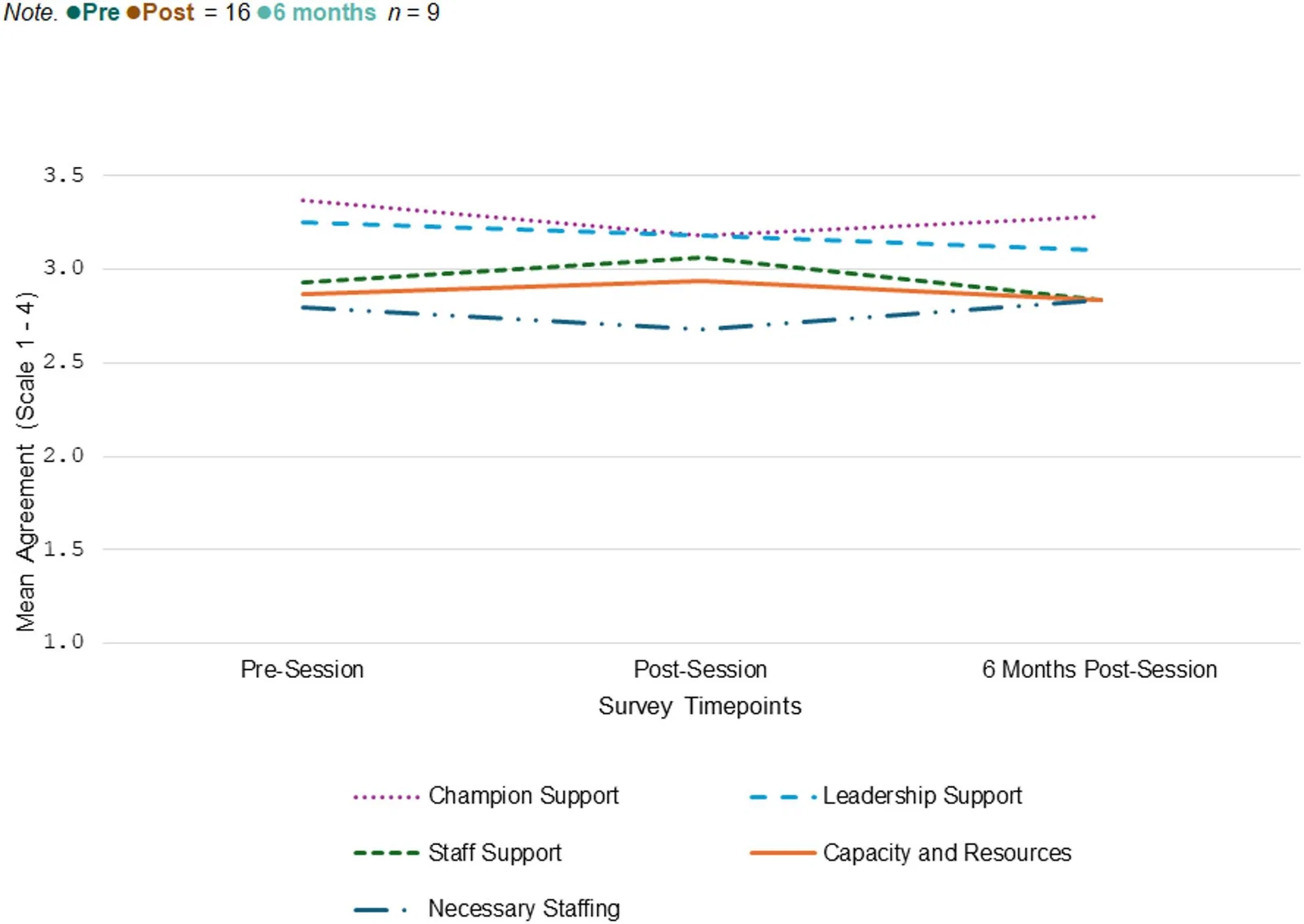
CRC screening program adoption facilitators at the organization-level. *N* = 16 matched from pre and post surveys. For the 6-month survey, 9 participants completed them, results were homogenized since the scale was slightly different

Multiple facilitators and barriers to CRC screening program adoption were identified across participating organizations. Adoption facilitators remained relatively consistent between the pre-ECHO (baseline) and 6-month post-ECHO surveys (Fig. 2). Key facilitators included CRC screening program champions, leadership support, staff support, and organizational capacity (e.g., adequate staffing, funding). Similarly, reported adoption barriers did not vary substantially between the pre- and post-ECHO surveys. Barriers included limited staffing, uncertain funding, and inadequacies in electronic health record (EHR) systems for patient identification and tracking (Fig. 3).

Program champions were widely recognized as critical but not consistently present across organizations. On a scale of 1 (strongly disagree) to 4 (strongly agree), mean agreement with the statement “My organization has one or more program champions,” declined slightly from 3.4 (baseline) to 3.3 ( 6-month). Importantly, program champions were widely viewed as clinic leaders who were appreciated and relied upon. Notably, champions were not limited to senior management roles and included providers and clinic managers.

Most organizations reported having leadership support for CRC screening patient outreach activities. Leadership was not limited to senior clinic leaders but also included clinic managers who were invested in the CRC screening program. Notably, limited leadership support did not preclude improvements in CRC screening activities. Findings suggest that leadership support stemmed either from personal investment in the success of the CRC program or from the need to meet external performance metrics, (e.g., coordinated care organization quality metric used to assess performance). Several organizations attributed a decline in leadership support to removal of CRC screening as a state-level Medicaid incentivized performance metric in 2020.

Adequate staffing was essential for an effective CRC screening patient outreach program. Some organizations hired dedicated outreach personnel responsible for identifying eligible patients for screening, conducting outreach calls, performing basic risk assessment, and referring higher-risk patients to providers. In organizations where hiring dedicated staff was not feasible, existing clinic staff were cross-trained, and patient outreach responsibilities were integrated into their existing roles.

Organizations shared that having an adequate EHR system capable of identifying and tracking patients was essential for an effective and efficient screening program. EHR capacity varied substantially, with some organizations reported robust systems and others identifying limitations in tracking screening eligible patients.

### CRC screening program modifications (Adaptation)

Participation in scale up activities led to modifications in existing CRC screening programs. 24% of participating organizations (*n* = 6) reported modifying SMARTER CRC program components (i.e., mailed FIT or patient navigation) because of their participation in the ECHO and other scale-up activities. 12% (*n* = 3) reported no planned modifications, and 8% (*n* = 2) were uncertain. The primary goal of modification was to improve screening rates and early detection. “We already have a colon cancer screening program implemented but wanted to learn what others are doing and make changes and improvements using best practices and leveraging other organizations’ successes.” (Participant ID 124).

Modifications were tailored to organizational context, including staffing, workflows, and patient population needs. Organizations shared that scale-up activities were an important source of information to identify CRC screening modifications. Modifications occurred at both organization and patient levels (Table 4).

**Table 4:** Organization and patient-level modifications

Type of Modification	Organizational Level Modifications	Patient Level Modifications
Patient Contact	Community health workers’ home visits include CRC screening patient outreach	• Customizing outreach activities to reach specific patient populations • Conducting patient outreach in multiple languages to be inclusive of all patients • Phone calls and CRC screening education information
Reminders	Increased frequency of patient communication	Use of multiple communication channels for patient communication • phone calls, text messages, letters, reminder cards
Tailored Communications	Providers communicate with patients, increasing awareness and knowledge of CRC screening	• CRC screening patient education tailored to specific patient populations • Tailored content and information delivered in multiple formats • text messages, social media, information sheets, clinic signage
Assistance	Providing patients with a chaperone to accompany them during their colonoscopy appointments	Providing transportation assistance to help patients travel from their home to their colonoscopy appointment
Overcoming Barriers	Creation of teams to facilitate CRC screening activities and resolve workflow processes and structural barriers to screening	Including a pen in the mailed FIT kit to improve completion rates

### Maintenance (sustainability)

Sustainability of CRC screening patient outreach activities was influenced by leadership support, staffing, EHR capacity, and funding. While many organizations expressed confidence in maintaining patient navigation activities, fewer reported stable funding for mailed FIT programs. Funding was a critical facilitator for both adoption and sustainability. More than half of organizations (56%, *n* = 8) reported confidence in having consistent financial support for mailed FIT programs, and a larger proportion (89%, *n* = 10) anticipated ongoing financial support for patient navigation. However, some organizations expressed concerns about long-term sustainability of mailed FIT and patient navigation programs citing the removal of CRC screening as a financially incentivized state-level metric [35]. Some organizations reported CRC screening and patient outreach activities were supported by grants. Grant funding enabled organizations to hire dedicated CRC screening staff and improve electronic health record systems. However, several organizations noted that reliance on grant funding to support a CRC screening program could limit sustainability, as funding was time-limited and could result in loss of program adoptions, modifications, and quality improvement gains [36–38]. 

## Discussion

This mixed-methods evaluation examined the effectiveness of a multi-level approach to facilitating health systems practice change aimed at improving CRC screening patient outreach in rural, low-resourced health care settings. Our primary outcomes included engagement in scale-up activities; adoption of SMARTER CRC patient outreach program components including mailed FIT and patient navigation; and modifications to program components. Overall, participating organizations were motivated to participate in scale-up activities to improve CRC screening rates, promote early cancer detection, and reduce CRC-related mortality within their communities.

Our findings suggest that the ECHO model was an effective component of a multifaceted strategy for scaling organizational knowledge and engagement in CRC screening patient outreach. Participants reported that the ECHO format, with its interactive structure and relevant content, facilitated learning. Engagement in ECHO increased awareness, generated new ideas, and provided participants with practical skills and resources to enhance CRC screening patient outreach. Importantly, organizations reported applying this knowledge to support their CRC quality improvement efforts and that the ECHO was a motivator for program expansion and quality improvement. This finding aligns with prior studies demonstrating the effectiveness of the ECHO model in building organizational capacity for quality improvement and disseminating best-practices in cancer screening and early detection, particularly in low-resourced primary care settings [33, 39, 40]. Collectively, this suggests that the ECHO approach can be a valuable mechanism for advancing knowledge and engagement in evidence-based CRC screening interventions in rural communities.

Our findings suggest that multicomponent strategies are effective for scaling up complex interventions in rural, low-resourced clinic settings, with the ECHO model serving as a key component supported by other strategies. In this study, the ECHO model proved to be an efficient and effective approach to scale-up the SMARTER CRC intervention when implemented in conjunction with other scale-up activities such as webinars and trainings. Consistent with recommendations from the Community Preventive Services Task Force (CPSTF), which endorse multicomponent interventions to increase CRC screening, our results indicate that a comprehensive strategy anchored by ECHO can successfully support spread of complex interventions in rural health care contexts [5, 41, 42]. Through six didactic ECHO sessions, participating organizations were exposed to a range of CRC screening strategies, including increasing patient awareness through targeted outreach (e.g., phone calls, texts, letters, reminders), implementing provider reminders, and integrating workflow processes to identify and track patients through the CRC screening process. Although participation in other scale up activities, such as patient navigation training, technical assistance, webinars, or a practice facilitation guide, was lower, these activities appeared to enhanced the overall impact of ECHO by offering additional support for organizations seeking more intensive capacity-building opportunities.

Engagement in the multifaceted scale-up activities influenced both the adoption and modifications of patient outreach activities, (e.g., mailed FIT and patient navigation). Participation generated ideas, provided practical skills, and shared resources, enabling organizations to clarify the objectives of their screening program and develop actionable plans to achieve short- and long-term goals.

Scale-up activities were more effective in facilitating program modifications to existing CRC screening programs than in fostering the implementation of entirely new screening programs. Although most organizations (83%, *n* = 21) were already conducting CRC screening activities at baseline, they used the knowledge and training gained through participation in scale-up activities to improve the reach and effectiveness of their existing CRC screening program through program modifications. Key modifications included designating dedicated CRC screening staff, ensuring adequate staffing for patient outreach, enhancing collaboration between the clinical and quality teams, improving EHR systems to identify and track patients, implementing annual mailed FIT campaigns, and addressing patient barriers by providing transportation from a patients’ home to the clinic. These findings suggest that structured, interactive scale-up activities can empower organizations in rural, low-resourced settings to improve existing programs and increase screening uptake. We collected data on modifications rather than adaptations because we found that organizations were more likely to make incremental changes in an ad hoc manner to improve their screening program, than follow a structured, deliberate approach. This finding underscores the practical, context-driven nature of practice change in rural, low-resourced settings.

Organizations emphasized the importance of patient-centered CRC screening, aiming to normalize screening, increase awareness of mailed FIT testing, and conduct patient outreach in multiple languages to reach diverse patients. CRC screening patient outreach adoption barriers and facilitators specified by participating organizations included adequate staffing, leadership support, CRC screening champions, workflow processes, and sustainable funding. All facilitators have been identified in prior research [43, 44]. Our results suggest that screening facilitators were highly dependent on the organization. For example, adequate staffing was a program facilitator in some organizations, while inadequate staffing was a noted barrier for other organizations. Organizations with limited staff resources for CRC screening outreach activities described their program as maintaining but were generally restricted in terms of adopting new screening activities. We found that the design and integration of workflows supported CRC screening programs in some organizations. Conversely, at some organizations, inefficient workflow processes limited the effectiveness of their screening program. Examples include inadequately identifying patients who are eligible for screening or having inadequate staffing to navigate patients with an abnormal FIT result to complete a follow-up colonoscopy.

## Limitations

Our study had some limitations we should mention. While each participant was provided a link to a Facilitators Guide, we were unable to track individual downloads of this resource, limiting our ability to assess the extent of engagement with this resource. Second, the sample size was relatively small, which may limit the generalizability of our findings to other rural, low-resourced clinic settings. Third, participating organizations were self-selected, introducing the possibility of selection bias. Our survey response rate of 19% overall is only slightly lower than the response rate for other ECHO follow-up surveys (24%, unpublished data). It is possible that organizations more motivated to improve CRC screening may have been more likely to engage in the scale-up activities and reply to the data collection requests. Finally, data on program modifications were self-reported and may be subject to recall bias. Despite these limitations, the study provides valuable insights into the role of the ECHO model and other scale-up strategies for CRC screening or other multi-level, complex interventions in rural, low-resourcedd healthcare settings.

## Conclusion

Participation in multifaceted scale-up activities increased organizational knowledge and engagement in CRC screening patient outreach within rural, low-resourced clinic settings, particularly for mailed FIT and patient navigation activities. Engagement in these activities also influenced the adoption and modification of CRC screening patient outreach strategies, with organizations more likely to make ad hoc modifications to existing screening programs than planned adaptations. These findings can inform future efforts to optimize scale up approaches for CRC screening and other complex interventions in rural, low resourced healthcare settings. 

## Supplementary Information

Supplementary Material 1.

Supplementary Material 2.

## Data Availability

This study collected and analyzed primary data. The authors have submitted the de-identified datasets to the Accelerating Colorectal Cancer Screening and Follow-up through Implementation Science (ACCSIS) Program data sharing website, which is a public repository.

## Abberviations

FIT: Fecal immunochemical test
ECHO: Extension for Community Healthcare Outcomes
SMARTER CRC: Screening More patients for CRC through Adapting and Refining Targeted Evidence-based Interventions in Rural settings
RE-AIM: Reach, Effectiveness, Adoption, Implementation, Maintenance
CSAT: Clinical Sustainability Assessment Tool
PSAT: Program Sustainability Assessment Tool
FRAME: Framework for Reporting Adaptations and Modifications – Expanded
FRAME-IS: Framework for Reporting Adaptations and Modifications to Evidence-based Implementation Strategies
OHSU: Oregon Health & Science University
