# Supplementary material for: Scale-up evaluation results of a multi-level approach using mailed immunochemical test (FIT) and patient navigation to increase colorectal cancer screening rates in rural clinic settings
Source: BMC Prim Care. 2026 Jul 14;27:361. doi: 10.1186/s12875-026-03428-w (PMC13656510; doi:10.1186/s12875-026-03428-w)
Supplement: Supplementary file 1 [file 12875_2026_3428_MOESM2_ESM.pdf]

SMARTER CRC
Improving colorectal health
in rural communities

Colorectal Cancer Screening Outreach for Rural Populations

Facilitation Guide

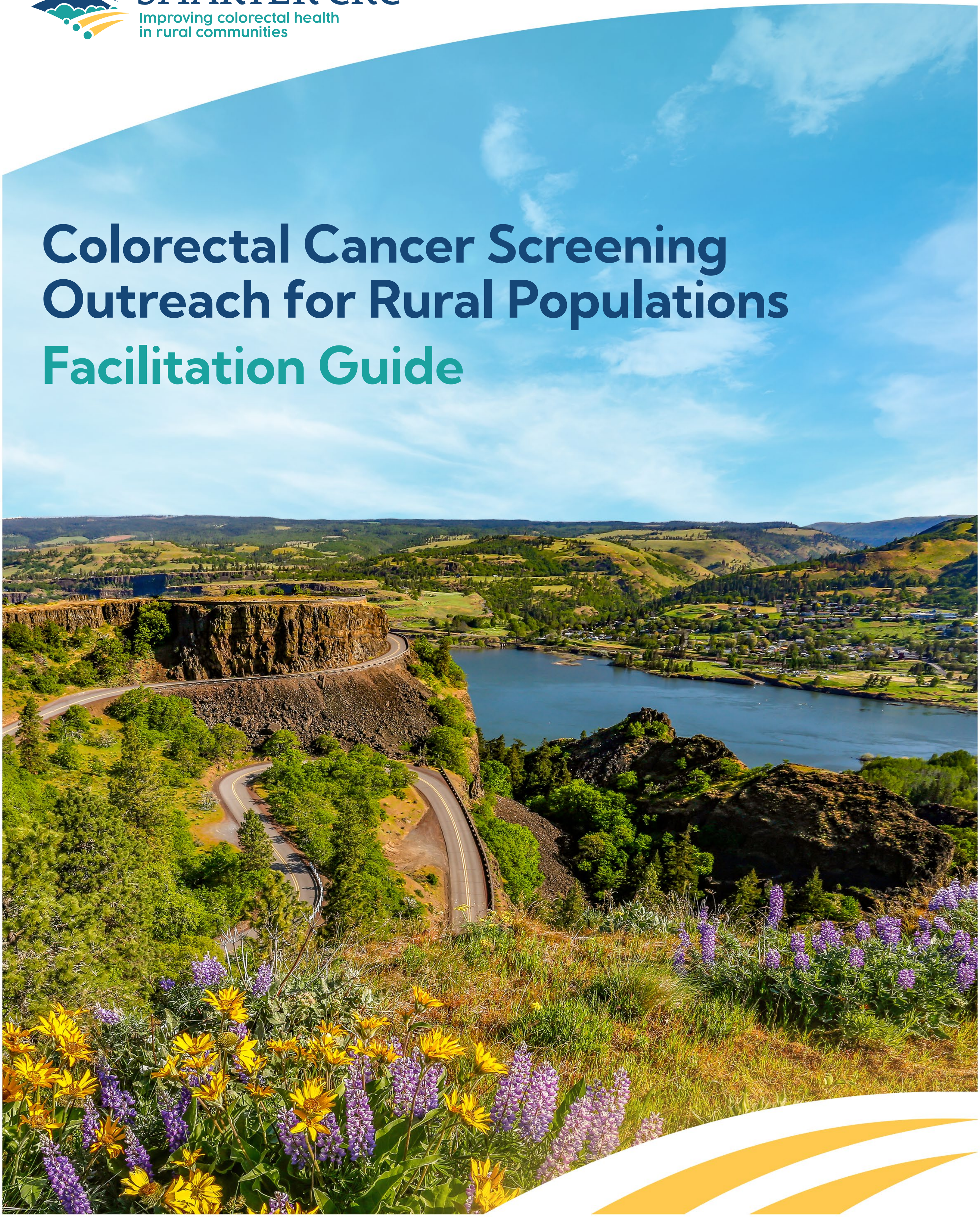

Acknowledgements

This project was supported by the National Cancer Institute of the National Institutes of Health under Award Number UH3CA244298. This work is part of the Accelerating Colorectal Cancer Screening and Follow-up through Implementation Science (ACCSIS) Program, a Beau Biden Cancer Moonshot[SM] Initiative. The overall aim of ACCSIS is to conduct multi-site, coordinated, transdisciplinary research to evaluate and improve colorectal cancer screening processes using implementation science strategies. The research team would also like to acknowledge our clinical practice and coordinated care organization research partners who were part of the SMARTER CRC study. The content is solely the responsibility of the authors and does not necessarily represent the official views of the National Institutes of Health.

Multiple Principal Investigators:

Dr. Melinda Davis
Oregon Rural Practice-based Research Network,
Oregon Health & Science University
3181 SW Sam Jackson Park Rd. Portland, OR 97239

Dr. Gloria Coronado
Kaiser Permanente Center for Health Research
3800 N Interstate Ave, Portland, OR 97227

This guide will provide a roadmap for how to implement colorectal cancer (CRC) screening outreach programs within your healthcare organization.

Delivering successful colorectal cancer (CRC) screening outreach in rural settings is a challenge on multiple levels. Compared to adults living in urban centers, adults living in rural and frontier communities have lower participation in CRC screening and follow-up care.

Project ECHO© (Extension for Community Health Outcomes) is a telementoring program that improves how health care is delivered to improve patient health outcomes and supports a wide range of topics. The materials compiled here complement the ECHO program on *Colorectal Cancer Screening Outreach for Rural Communities.* The ECHO will walk through ways to promote CRC screening in various health care settings. It will focus on two approaches strongly supported by research: mailed fecal test outreach and patient navigation to follow-up colonoscopy. In this guide, you will find background information and links to resources about options for screening outreach, types of testing, cost effectiveness of screening outreach, and implementation strategies for mailed fecal immunochemical testing (FIT) and patient navigation, such as patient identification and templates for clinical practice workflow design.

ECHO LEARNING SESSIONS: COLORECTAL CANCER SCREENING OUTREACH FOR RURAL COMMUNITIES

1. What is the Business Case for Colorectal Cancer Screening Outreach?
2. Building Engagement for a Collaborative Colorectal Cancer Screening Program
3. Designing a Mailed FIT (Fecal Testing) Program that Works for your Organization
4. Identifying your Patients due for Colorectal Cancer Screening
5. Patient Outreach and Communication: Kit Mailing, Reminders and Follow-up
6. Navigating Patients to Follow-up Colonoscopy

SMARTER CRC
Improving colorectal health in rural communities

CRC Screening Outreach Facilitator's Guide: Introduction

Purpose

Colorectal cancer (CRC) is the second-leading cause of cancer deaths, and early detection saves lives. When colorectal cancer is detected early, the chance of surviving five years is 90%. Survival drops to 69% for cancers that are diagnosed at regional stages, and to 8% when found at an advanced stage.

Delivering successful CRC screening outreach in rural settings is a challenge on multiple levels. Compared to adults living in urban centers, adults living in rural and frontier communities have lower CRC screening and follow-up rates. This facilitator guide is one component of The *Colorectal Cancer (CRC) Screening Outreach for Rural Communities* program:

- **An ECHO series** (https://www.oregonechonetwork.org/programs) offered in Spring and Fall 2023 that walks through ways to raise rates of CRC screening. It will cover how to design a program that could involve a partnership between clinical practices and regional organizations to CRC screening outreach.
- This **CRC Screening Outreach for Rural Organizations Facilitator's Guide** with self-assessments, links to background evidence supporting CRC screening interventions, and templates and check lists for implementation.
- Access to **technical assistance** from a variety of experts who have conducted research and outreach programs across clinical and community settings.
- A virtual **patient navigator training** for navigating patients to colonoscopy.
- **Expert resources and webinars** on a variety of related topics that are referenced throughout the ECHO series and the Facilitator's Guide.

Audience

This facilitator's guide is intended to be used by Organizational Facilitators who will be leading implementation of the program in health care or community organizations (health plans, CCOs, health or clinics systems, or multi-site clinical practices) interested in improving CRC screening rates using a population outreach program.

Program leads are encouraged to create a cross-functional team as relevant to their organization. Cross-functional teams might include: quality improvement leaders, population health specialists from health plans or clinical systems, clinic administration staff, health insurance plan staff, Medicaid population health specialists, community health workers, provider champions, medical assistants, or nurse managers.

Facilitation Guide: Contents

This facilitator's guide is meant to be used as a companion guide to the ECHO series on *Colorectal Cancer Screening Outreach for Rural Communities*. These materials are aimed at the organizational facilitators who are part of a team implementing a colorectal screening outreach program. The following pages list the Sections and related materials for each step.

Definitions:

FIT: fecal immunochemical test

CRC: colorectal cancer screening

ECHO: Extension for Community Healthcare Outcomes telementoring program

PLANNING

Facilitator's Guide: Section 1. Organizational Assessment and Pre-implementation Planning

This section provides an outline to conduct an initial assessment as your organization starts the ECHO series on *CRC Screening Outreach for Rural Communities* plus an overview of why you might outreach to patients.

Topics included:

- Organizational assessment to prepare for CRC screening outreach
- Business case for CRC screening outreach strategy, including program costs and return on investment for mailing outreach
- Overview of mailed FIT outreach and patient reminders and follow-up

SMARTER CRC
Improving colorectal health in rural communities

DESIGNING YOUR PROGRAM

Facilitator's Guide: Section 2. Design your CRC Outreach Program

This section provides information on decisions you will need to make as the organizational lead when designing your CRC screening outreach program, including the overall approach, the team, whether to involve vendors to accomplish some steps, and how to run a collaborative program with buy-in across organizations.

Topics included:

- Building your organizational team, choosing your patient population, engaging clinical practices and health systems
- Choosing your implementation strategies
- Choosing your fecal immunochemical test (FIT)
- Working with a vendor for sending kits and/or reminders
- Establishing a Learning Collaborative structure with participating clinical practices

Resources/Materials

- Agenda samples for each Learning Collaborative meeting and slide templates
- American Cancer Society's fecal immunochemical test (FIT) selection guide (https://nccrt.org/resource/fobt-clinicians-reference-resource/
- Handout for providers to learn about the options for CRC screening

WORKFLOW ASSESSMENT

Facilitator's Guide: Section 3. Outreach Program Workflow Assessment

This section provides guidelines for how to operationalize a multi-organization collaborative program that is adapted for implementation in a clinical practice environment.

Topics included:

- Instructions for meeting with implementing organizations and using a Workflow Assessment Guide to create an outreach plan for a health plan, health system, or clinical practice
- Developing a swim lane workflow diagram

Resources/Materials

- Clinical Practice Workflow Assessment Guide
- Workflow example for a large system clinic
- Workflow example for clinics, health systems, or health plans that centrally process their FITs
- Workflow example for a small rural clinic

GENERATING YOUR ELIGIBLE PATIENT LIST

Facilitator's Guide: Section 4. Generating your Eligible Patient List

This section provides guidelines for how to identify individuals who are due for CRC screening using a combination of claims and EHR data.

Topics included:

- Generating a patient list and tracking CRC screening outreach and follow-up events.
  - Prioritizing populations (prior screened, new screeners, 45-54 year-old patients)?
- An overview of clinical practice review of a health plan generated list (i.e., "scrubbing")

Resources/Materials

- Data request samples for an eligible patient list
- Handout and training slides for chart scrubbing
- Templates for tracking eligible patient lists (www.mailedfit.org)

MAILING OUTREACH & REMINDERS

Facilitator's Guide: Section 5. Mailed FIT Outreach

- An overview of best practices for a Mailed FIT program, including prompt and reminders
- Strategies for addressing rural-specific challenges to mailing programs
- Strategies for tracking FIT returns, lab order processes, and lab results

Resources/Materials

- Prompt (Prior to the Mailing) Call/Text Script
- Introduction Letter, customizable bilingual (English/Spanish) letter to use prior to mailing FIT kit
- Kit Insert Letter, customizable bilingual (English/Spanish) letter included with the FIT
- Frequently Asked Questions, bilingual (English/Spanish) for call center or front desk staff to use in conjunction with FIT mailings
- Reminder Call Script that can be used after a FIT mailing to remind patients to return FITs

PATIENT NAVIGATION

Facilitator's Guide: Section 6. Patient Navigation

This section provides an overview of a Patient Navigation program and instructions for using the Patient Navigation Workflow Assessment Guide to define how a clinical practice or health plan delivers navigation to colonoscopy for eligible patients.

Topics included:

- Patient navigation overview
  - Why use patient navigation to address barriers to colonoscopy?
  - Core elements of a successful patient navigation model
  - Patient navigation protocol and adaptations for a rural setting
- Distributed vs. centralized models of navigation staffing

Resources/Materials

- Patient Navigation Training Session: Teaches navigators how to guide patients to get a colonoscopy (either as a follow-up to the FIT or as a screening colonoscopy).
- Patient Navigation Workflow Assessment Guide
- Patient Navigation one-page guides (1. First Call and Barrier Assessment, 2. Bowel Prep Review, 3. Colonoscopy Check-In, 4. Colonoscopy Results)

SECTION 1

Organizational Assessment and Pre-implementation Planning

This section provides an outline to conduct an initial assessment as your organization starts the ECHO series on *CRC Screening Outreach* and an overview of why you might implement a mailed outreach strategy.

TOPICS INCLUDED

1. Organizational assessment to prepare for CRC screening outreach
2. Business case for CRC screening outreach strategy, including program costs and return on investment for mailing outreach, patient response to mailed FIT
3. Overview of an intervention strategy: mailed outreach and patient reminders and follow-up

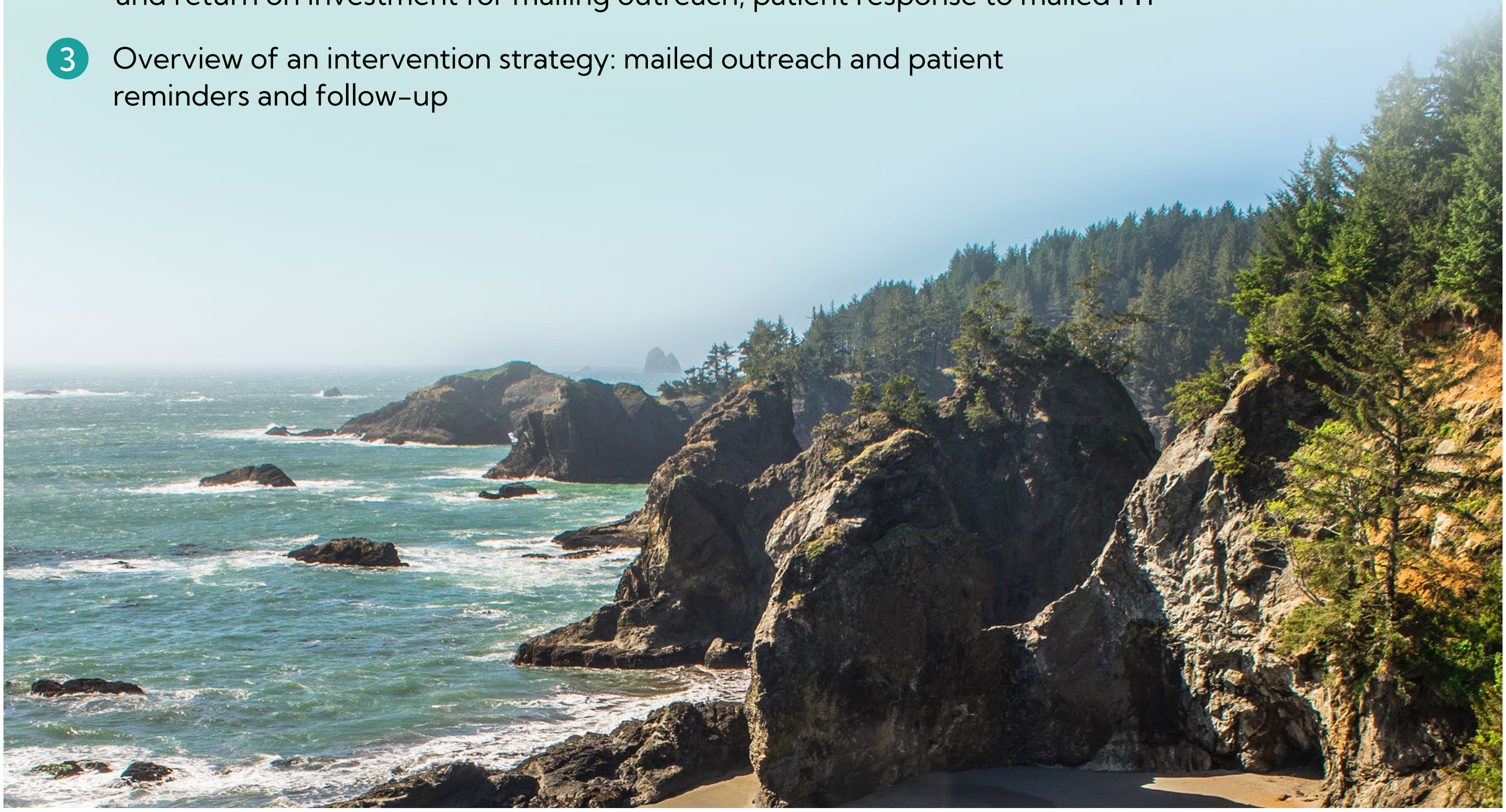

1.1. Organizational Assessment to Prepare for CRC Screening Outreach

The worksheet below is a guide for assessing current activities at a health plan or other health care organization and then making key decisions about CRC screening outreach. Answering these questions will clarify how your organization takes the recommended steps as you establish and develop a collaborative CRC screening intervention with participating clinical practices. *The table outlines key decisions points for health plans or other organizations considering a screening outreach program.*

Health System or Organizational Experience with CRC Screening Work Sheet

Assessment	Answers
**PREPARE: Current Screening Efforts and CRC Outreach**	
What is your health organization or health system doing now to make sure eligible patients are screened for colorectal cancer (CRC)? What are the current practices in your organization related to getting people screened for colorectal cancer (CRC)?	
Is your organization already using evidence-based approaches (such as navigating patients to screening, outreach using fecal tests, reviewing patients coming in for visits) to screen patients for CRC? If so, what are they?	
What has worked and not worked in the past when your organization has tried to increase CRC screening?	

Health System or Organizational Experience with CRC Screening Work Sheet (Continued)

Assessment	Answers
What has worked and not worked in the past when your health care organization has tried to increase screening for other diseases, such as breast cancer, cervical cancer, or diabetes? Are there lessons learned that can be applied to CRC screening?	
Will you want to conduct an outreach all at once to your whole population, or start with a sub-set of the population or clinical practices? Will you want to do an opt-in program for clinical practices?	

Business Case for CRC Screening Outreach

If you are reading this guide, likely you already have interest in learning more about outreaching to patients to complete CRC screening. One method in particular that is effective is running a direct-mail fecal immunochemical test (FIT) program, which can help to overcome many common screening barriers around time and transportation logistics, disparities, and inconveniences. Mailed FIT outreach kits offer a convenient, at-home, minimally-invasive alternative to colonoscopy.

To launch and sustain a CRC screening outreach program, such as a mailed FIT program, start with gaining leadership and organizational support. Some systems, leadership, and even individuals are not "sold" on implementing FIT programs. Getting support at ALL levels is very important for a program to be successful and sustained. Think about the value proposition of colon cancer screening:

- Cost of a single FIT test and processing: ~$12-35
- Cost of treating a single case of colon cancer: ~$46,000

Argument #1: Screening saves lives

- For the cost of treating 1 person with colon cancer, your program can perform 3,833 FIT tests.
- And if you were to perform 3,833 FIT tests, you would find an estimated 10 colon cancers. (BUT also many polyps would be removed that never turn into cancer.)

Argument #2: FIT programs are cost-saving because they reduce the need for expensive colonoscopies.

- FIT is as effective for a portion of the cost, and screens more people, with better results.
- In a study done by the CDC, the cost per person was $3,153 for programs using colonoscopy and $1,291 for those using FOBT/FIT with diagnostic colonoscopy following FIT completion

Argument #3: FIT tests are more acceptable for patients

- FITs are easy to do.
- FITs can be done at home.
- Colonoscopies can be risky (including potential hospitalization, perforation, major bleeding, diverticulitis, cardiovascular events, and/or serious abdominal pain).
- Patients screen more when they can make a choice about their screening options.

Argument #4: Programs can become more affordable through cost-sharing partnerships and optimizing outreach approach

Partnerships with other organizations can help creating cost-sharing opportunities in a mailed FIT program. The approach we present throughout this facilitator's guide involves a health plan or other central organization paying to mail-out FITs to their population and clinics tracking and processing those tests, according to standard care. You can also work with community organizations, organizations such as American Cancer Society, and even private donors and foundations to cover the costs of the FIT mailings. Finally, state or federal incentives for CRC screening can offset the cost of outreach programs, so be sure to reach out to your legislators and encourage them to incentivize CRC screening. In addition, as we will cover in the cost section below, carefully planning where and how your FIT kits are processed has cost and reimbursement implications.

Cost and Return on Investment for Programs

Fecal Immunochemical Tests (FIT) and Lab Processing

Because CRC screening is recommended by the U.S. Preventive Services Task Force, FITs are covered under the Affordable Care Act. Therefore, the lab processing for FITs is covered by the typical insurance claim mechanism (such as a Medicaid or Medicare claim). In many cases, FITs can be acquired for free; many laboratories provide FITs to clinic systems for free because they will be paid when the tests are returned for processing.

In-house processing of FITs can make the program more financially sustainable. If your clinic system or organization is processing FITs in-house, you need to calculate how much it costs to purchase and process the tests. However, if a lab is located within your organization (i.e., in-house), you will receive the reimbursement to cover the costs of the FIT test and processing for any insured patients.

Costs		Reimbursement	
Cost of FIT test + mailing	$8	Reimbursement per test from insurance	$23
Cost of FIT processing by in-house lab	$6		
**Total**	**$14**	**Total**	**$23**

If you get a 50% return rate (and you can bill for all your patients), your program would pay for itself. If you have a high uninsured population or a low response rate to a mailed outreach, then you need to think about other sources of funding for the program.

Printing, Preparing Materials, and Mailing

The largest costs of the program outside of labor are the printing, preparing FITs for mailing, and postage costs. All health plans in the SMARTER CRC research trial contracted with a vendor to conduct these tasks. Below are estimates on the cost of printing and preparing an introductory letter sent before the FIT mailing, and the subsequent FIT kit with instructions and an explanatory letter. Strategies to reduce costs include printing in black and white or removing the introductory letter. However, postage is typically the costliest line item.

To reduce postage costs, a good practice is to set up a Business Reply envelope linked to your organization. You can cut costs by only paying for return postage for the kits that a person completes (instead of putting postage on all outbound reply envelopes). In addition, some practices prefer for patients to physically bring a kit into a clinical office instead of mailing it back. The sample costs below cover outbound mailing and printing costs using a mailing vendor to prepare the kits.

FIT Outreach Program: Printing and Mailing Costs*

Mailing and printing<br>Number of FITs ~565-590	Costs
Activities included:<br>• Setup projects, purchase and prepare envelopes<br>• Prepare and mail introduction letters<br>• Prepare and mail FITs including printing instructions, letters, and labeling individual FITs with patient information	$ 2,930.00 – $ 3,680.00
Outbound Postage	$ 6-8 / each depending on FIT test

** Costs are based on program implementation by two Oregon health plans in 2021.*

Cost of the actual FIT tests are not included above because, for this FIT mailing program, they were mostly provided free from the labs who would process the FITs and receive reimbursement. The average cost per patient who returned FITs varied based on postage costs for the reply postage by FIT type and differing response rates. Costs wound up being about $ 6-13 / each at about a 15% response rate in the first year of the program. Typically response rates are higher in the second year and the per person cost will be lower in the second year of a program.

Patient Navigation and Labor Costs

Labor is the costliest aspect of an outreach program. Several of our research collaborators applied for grants to cover a staff member who was focused on preventive care outreach and this became part of their activities. The *Steps for Increasing Colorectal Cancer Screening Rates: A Manual for Primary Care Practices* (https://nccrt.org/resource/steps-for-increasing-crc-screening-rates-2022/) has a nice set of resources for applying for grants to cover staffing for outreach, especially for a patient navigator. And see also the National Cancer Institute Health Patient Navigator Program (https://www.cancer.gov/about-nci/organization/crchd).

A few academic manuscripts have studied overall costs of FIT mailing and patient outreach programs. These studies found:

- From the perspective of state Medicaid system, a patient reminder + mailed FIT was more cost-effective than a screening reminder-only alternative, with lower costs and higher screening rates.[1]
- A study of two different approaches to implementation of a Medicaid/Medicare health plan mailed FIT program in 2016 showed development costs were $23.0K, primarily administration, in one model, and were $37.3K, 38.8% for FIT selection and mailing/tracking protocols in the second model. Implementation costs were $18.36 and $16.34 per enrollee respectively for the two programs.

In addition to the costs of FIT mailing, the addition of a trained patient navigator fills an important role as follow-up after the FITs are returned by patients. This part of the program is discussed in detail in Section 6, but briefly this person calls eligible patients to help "navigate" them towards getting screened for CRC with the goal of overcoming any barriers to screening, and to support diagnostic testing and treatment initiation. See also the American Cancer Society article for a nice summary.

A look at cost-effectiveness of a patient navigation intervention to increase colonoscopy screening among low-income adults in New Hampshire found:

- Navigated patients are more likely to complete screening and less likely to cancel on the day of the appointment or to miss their appointment. Investing in patient navigation leads to cost savings by reducing the loss of revenue from missed appointments and late cancellations that remain unfilled.
- Patient navigation improves the patient experience, which has the potential to reap longer-term financial rewards because patients are more likely to return to the provider or recommend him or her to others.
- The incremental cost of a patient completing a colonoscopy with patient navigation was $548 under the endoscopy center scenario and $725 under the public health program scenario. The average cost of providing colonoscopy alone was $737, leading to cost savings of $189 and $12, respectively, under the endoscopy center and public health program scenarios.

Another study looked at different simulated interventions and estimates of effectiveness and cost and found the following:

- The cost per patient of mailing a FIT directly to the patient's home was $20.84 (Smith, 2012).
- The cost per patient for a trained patient navigator to call eligible patients to "navigate" them towards getting screened for CRC was $1159.64. (Smith et al., 2012; Lairson et al., 2014; *Patient Navigator Training Collaborative*, 2019; Freeman, 2019)
- The cost per patient of a *combination* of mailed FIT and navigation, with navigators focused on FIT completion or follow-up after an abnormal FIT $1180.48 (Smith, 2012; Lairson et al., 2014; *Patient Navigator Training Collaborative*, 2019; Freeman, 2019)

Citations

[1] Wheeler et al. Comparative cost-effectiveness of mailed fecal immunochemical testing (FIT)–based interventions for increasing colorectal cancer screening in the Medicaid population. Cancer. 2020 Sept;126(18):4197–4208. Epub 2020 Jul 20.PMID: 32686116. https://acsjournals.onlinelibrary.wiley.com/doi/full/10.1002/cncr.32992

[2] Meenan RT et al. Costs of Two Health Insurance Plan Programs to Mail Fecal Immunochemical Tests to Medicare and Medicaid Plan Members. Popul Health Manag. 2021 Apr;24(2):255-265. Epub 2020 Jun 29. PMID: 32609077. https://www.liebertpub.com/doi/10.1089/pop.2020.0041

[3] Davis MM, Nambiar S, Mayorga ME, Sullivan E, Hicklin K, O'Leary MC, Dillon K, Hassmiller Lich K, Gu Y, Lind BK, Wheeler SB. Mailed FIT (fecal immunochemical test), navigation or patient reminders? Using microsimulation to inform selection of interventions to increase colorectal cancer screening in Medicaid enrollees. Prev Med. 2019 Dec;129S:105836. doi: 10.1016/j.ypmed.2019.105836. Epub 2019 Oct 18. PMID: 31635848; PMCID: PMC6934075.

[4] Rice K, Sharma K, Li C, Butterly L, Gersten J, DeGroff A. Cost-Effectiveness of a Patient Navigation Intervention to Increase Colonoscopy Screening Among Low-Income Adults in New Hampshire. Cancer. 2019 Feb 15; 125(4): 601–609. Published online 2018 Dec 12. doi: 10.1002/cncr.31864

[5] US Cancer Statistics Working Group. United States Cancer Statistics: 1999–2008 Incidence and Mortality Web-based Report. Atlanta, GA: Centers for Disease Control and Prevention, US Dept of Health and Human Services and National Cancer Institute; 2010. http://www.cdc.gov/uscs.

Additional resource: https://www.cdc.gov/cancer/crccp/field-guide/

CRC Screening Intervention Strategy: Overview

The following diagram outlines the steps in an intervention strategy using mailed FIT outreach and reminder processes, including navigating patients to follow-up colonoscopy. The ECHO learning series and this collection of resources are designed to walk you through setting up a program like this with one or more participating health care organizations, such as clinical practices, health insurance plans, or hospital-based systems. The results of a research study presenting outcomes and learnings about how to implement such a strategy are being published now.

What Did We Study?

IDENTIFY ELIGIBLE PATIENTS

Health plan generates list of eligible Medicare patients

Clinical practices "scrub" list for accuracy

PATIENT PROMPT & MAIL FITS

Vendor mails FIT kits

Clinical practices conduct a patient prompt (phone call, text message, letter)

RECEIVE FITS & PATIENT REMINDERS

Patients receive FITs

Clinical practices or health plan conduct reminders to encourage patient completion of test

PATIENT NAVIGATION

Navigate with abnormal FIT for follow-up colonoscopy

SECTION 2

Design Your CRC Outreach Program

This section provides information on decisions you will need to make up front as the organizational lead for a CRC screening outreach program, including the model of implementation, the team, whether to involve vendors to accomplish some of the logistics, and how to run a collaborative program with buy-in across organizations.

TOPICS INCLUDED

1. Building your organizational team, engaging clinical practices and health systems
2. Choosing your implementation strategies and your patient population
3. Choosing your fecal immunochemical test (FIT)
4. Working with a vendor for implementation
5. Establishing a Learning Collaborative structure with participating clinical practices

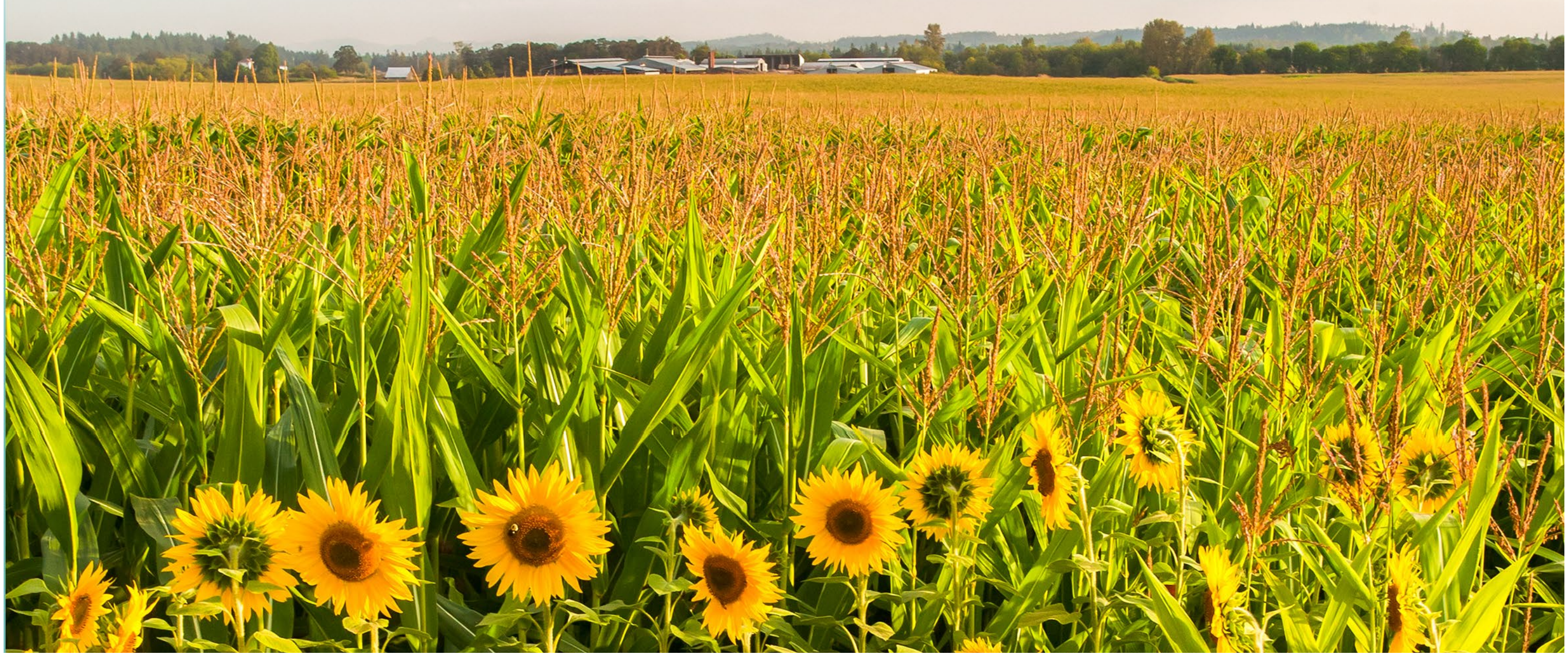

Building an Organizational Team

As with all quality initiatives, you will want to develop a core team up front familiar with the overall goals of the outreach. Your team will include members from all the organizations involved in a collaborative partnership to deliver screening outreach. Here's a guide to who might be on your team. Please note that every organization and approach will be different so this composition will vary.

Some organizations may decide to go with a centralized approach and others with a more distributed approach. Either model will work. The most important thing is to have a core leader in charge of facilitation and coordination across organizations and clear communications involving everyone at an organization that might be impacted. You will also want a *clinical practice champion*—someone who is trusted by leadership, enthusiastic about colorectal cancer screening and the program, and can be a motivator for others.

Staff Involved	Roles & Activities	Effort Level/Time Commitment
Project Lead (at a health plan or health system administrative level)	Overall lead for the project to facilitate the implementation and work with the collaborators in participating organizations	HIGH
Champion (may or may not be the same person as the Project Lead; likely this person would be at the clinical practice levels)	Set up introductory meetings to discuss the importance of CRC screening and the approach, get buy-in from providers and clinical practice staff, work on planning and policy development, smooth the implementation of the program	HIGH
Clinical Practice Manager/ QI or Population Health Manager	Ensure the correct practice-level staff are involved in planning, coordinate with lab managers and IT staff as needed,	HIGH
Medical Assistants and Referral Coordinators	Review or "scrub" the eligible patient list and remove patients who should not receive outreach, place FIT lab orders, receive FITs back in clinic, answer patient questions, update the EHR as needed	MEDIUM
Patient Navigator	Conduct outreach to patients for follow-up to FIT or for screening colonoscopies when required	MEDIUM
Community Health Worker, Medical Assistants	Patient outreach calls (if conducting non-automated prompts or reminders to return FITs)	HIGH/MEDIUM

Staff Involved	Roles & Activities	Effort Level/Time Commitment
Health Plan Data Analyst/Clinical Practice EHR Specialist	Reporting features can make population identification or review at the clinical practice level much more efficient.	MEDIUM
Clinical Practice Lab Manager/ Coordinator	FITs are typically ordered and processing is coordinated at clinical practice labs so this person will need to arrange to order FITs, be aware of the program, and be ready to receive FITs returned by mail. If using a central FIT (see below) this role is not typically involved.	LOW
Providers	Provide usual patient care and need to understand the basics for CRC screening modalities and program approach	LOW
Call Center or Front Desk Staff	If FITs are not returned directly to labs, these staff might receive and send along FITs, or the might be involved in answering phone calls	LOW

Examples:

- Organizational Champion (HIGH): This person likely needs about 1–2 hours per week in the initial stages to meet with different partners, however less time is needed in later stages.
- Medical Assistants/Referral Specialists (MEDIUM): If your organization chooses a collaborative review of the patient lists, the Eligible Patient List Scrub will take about ~20 Hours/100 patients. The rest of the effort would be encompassed by processing returned FITs and recording the information in the medical records if this is not automatically done via lab interfaces. This example shows how the approach you take can drastically change your staffing model at clinical practices.
- Providers (LOW): The advantage of centralized population outreach is that providers are not directly executing program activities, but should be aware of the program and understand its importance.

Engaging Clinical Practices and Health Systems

Once you have your core team established, you will want to engage with collaborating clinical practices or other outreach partners. When engaging a system, ensure that the participating clinical practices or other entities who will help implement your outreach program (such as a lab collaborator) are all on board and that messaging is clear on expectations. It is always better to notify and inform extra people who might be affected rather than assuming it will not impact their operations. In terms of gaining buy in from your collaborators, here are some common questions that might be raised or reasons an organization does not want to participate, along with strategies to address those questions.

Questions to Address with Outreach Partners	Solutions, Selling Points, and Strategies
General questions of readiness to implement an outreach program, such as lack of IT infrastructure to produce needed data	Highlight that CRC outreach can be done with incremental changes and make sure your program can support partners with less data infrastructure.
Worried about too many referrals to colonoscopy and not enough capacity if a FIT mailing is implemented	Using FIT testing can actually relieve pressure from colonoscopy providers by using resources for follow-up colonoscopies and removing the need for some general screening colonoscopies.
Not enough staffing and clinic capacity; "we don't have time for this"	Stress that a collaborative program can take something off the plate of the providers by centralizing the effort of outreach within your organization; also address staffing needs with support staff for the program, such as navigation services or reminder phone calls.
Worried about potential cost to the clinical practices and cost to the patients	Make sure that the clinical practices do not have to bear the burden of the costs, such as FIT tests, mailing, or lab processing. Ensure that billing is structured correctly so that patients do not receive bills for either FIT processing or follow-up colonoscopy.
Do not have a lab that processes FIT test that could work with a mailed FIT	Programs can be defined in a way that enables collaboration with many types of labs; refer to the *Selecting a High-Quality FIT Section* for more information.
Not a priority for their clinic; they have other health measures to address that are more urgent within limited bandwidth (for patients and providers)	If you have resistance to the idea, start the program with more receptive clinics or pilot the program with a smaller sample (see below for the opt-in strategy of program collaboration). Ensure the program is collaborative with other clinic activities.

Questions to Address with Outreach Partners	Solutions, Selling Points, and Strategies
They have other CRC outreach activities in a clinical practice already and are worried the FIT mail out will go to patients already getting a FIT	Ensure smooth collaboration by ample communication with providers and other programs (i.e., health plan) that work with patients on CRC screening; equip staff with messaging to help address individual questions from recipients of a mailed FIT.
Some communities might be more resistant to the idea of outreach due to cultural barriers.	Have some way to customize outreach to fit what works for that community, such as ensuring materials and outreach methods are culturally appropriate.

Key Question: Will your organization roll-out the program for all clinical practices or use an opt-in to the program model?

Include All Practices

Pro: Including all clinical practices can have less administrative burden and larger effect in terms of outreach.

Con: If a person receives a FIT from your organization and the clinical practice has not agreed to be a part of the program, they might be caught off guard. You might not be targeting your program to the people who need it the most.

Opt-in Programs

Pro: Clinical practices that have opted into a program might be able to collaborate on patient reminders, patient support with questions, and promotion of CRC screening overall. They will at a minimum be aware of the program if one of their patients asks about a FIT they receive in the mail.

Con: There's more administrative complexity. The program might not reach as many people if clinical practices do not opt into the program.

Regardless of overall approach, your organization will want to engage the participating clinical practices to discuss implementation and participation. Take a look at the different implementation strategies listed in the table below, and decide how much of these decisions your organization, as the lead, would need to make in advance of engaging partner organizations or if you would like to have the collaborating organizations help select implementation strategies. Either way, you will want to do the following to engage partners:

- Meet with clinical practices to introduce the program and make the case for CRC screening outreach.
- Engage a champion at the clinical practice level.
- Get clinical practice buy-in to help determine the implementation strategies or to support the screening outreach program elements (see decision making table below).
- Get clinical practice buy-in for providing follow-up and colonoscopy referrals to individuals being screened. Collaboratively review program elements and define roles (what is the lead organization doing vs. practice-level staff?).

Implementation Strategies

Organizations have a lot of strategies for increasing rates of CRC screening in populations. Approaches can take a wide range of activities, such as:

- Coordinated team-based response in clinical practices to identify patients due for CRC screening when they come in for appointments and refer them to colonoscopy or hand them FITs to take home and complete. (client/provider reminders)
- Provider-based performance interventions (provider assessment and feedback)
- Outreach calls or EHR reminders that provide information about screening more generally or remind patients due for screening to complete the provided tests or come in for screening (client reminders)
- Mailed FIT tests to patients due for screening at a population level (reduce structural barriers)

Provider Assessment and Feedback	Provider Reminders	Client Reminders	Reducing Structural Barriers
Assessing individual provider performance & comparing to other providers	Activating/utilizing EHR provider reminder function	Telephone or print reminders	Reducing the time or distance between patients and screening
Fostering competition by publishing screening rates of "competing" providers or clinics	Creating a system where reminders are clearly visible on paper charts or for office visits	Information about screening and ways to overcome barriers to screening	Simplifying administrative procedures and other obstacles

This guide will focus on a multi-level intervention for mailed FIT outreach combined with patient navigation to colonoscopy that addresses several of the categories listed above. Much of the information included here can be applicable to many different types of outreach. For example, identifying the population eligible or due for CRC screening would apply to many types of screening improvement efforts. See *The Community Guide* for additional information about multicomponent interventions not included in this guide if your organization is interested in exploring more options.[1]

In particular research has shown that *combining* multiple approaches at once leads to higher screening rates.[2] Ideas such as setting goals or structures based on evidence-based interventions can be multi-faceted, fulfilling more than one of these categories at a time. Therefore, combining 'client reminders', such as a patient reminder call, with 'provider reminders' such as an EHR flag at the time of an appointment, could remind the provider to offer CRC screening options. This multi-component intervention can help to enhance screening outcomes.

Other guides have a lot of great information about logistics, especially on running a mailing outreach program. These guides are aimed at the clinical practice level, and we are focused on sharing resources for a more collaborative outreach approach. In particular, the following two guides have a lot of great practice-level advice and would be well worth reviewing:

- **National Association of Chronic Disease Directors: Mailed FIT Implementation Guide** https://chronicdisease.org/using-the-mail-to-help-save-lives/
- **Steps For Increasing Colorectal Cancer Screening Rates: A Manual For Primary Care Practices** https://nccrt.org/resource/steps-for-increasing-crc-screening-rates-2022/
- **The National Association of Chronic Disease Directors has a self-paced video training series about Mailed FIT Testing** that is an excellent complement to the content in this facilitator's guide: https://chronicdisease.org/page/mailed-fit/

Implementation Strategies: Mailing, Reminders, and FIT selection

The mailing and patient reminder process has several key decisions that need to be made along the path to implementation.

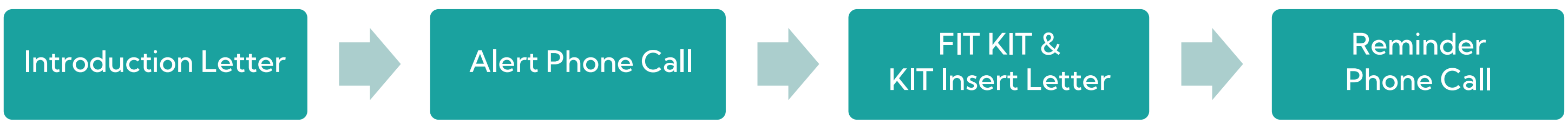

Central or Distributed FIT returns

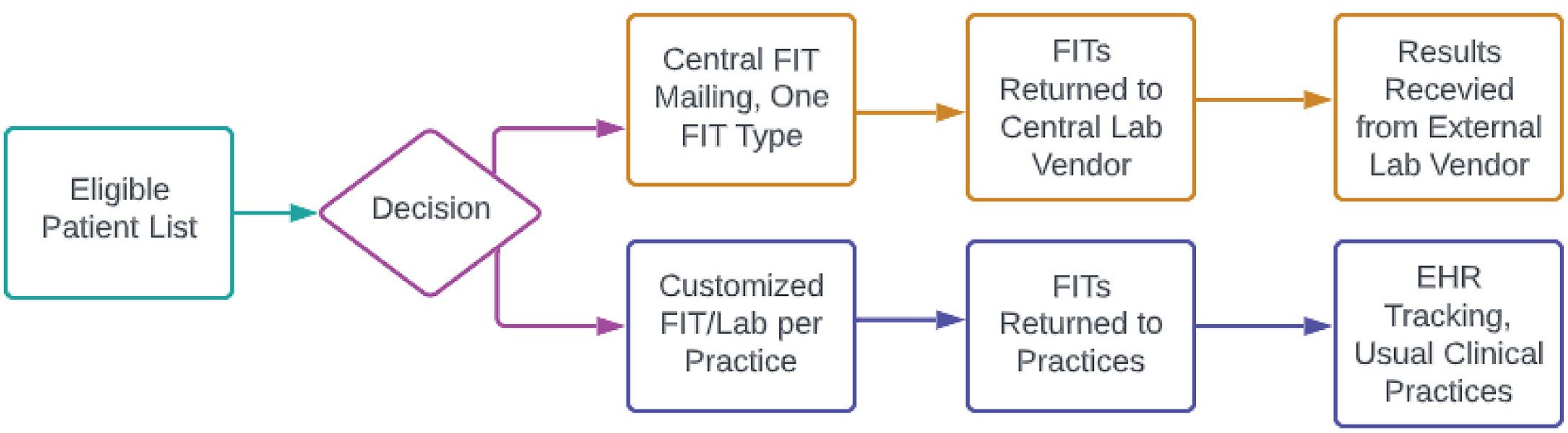

The diagram above shows a few example decisions. The answers to the questions in the following table will affect how your organization will proceed with a mailed outreach. Every organization has a different context, so there are not right or wrong answers, just different considerations.

Implementation Strategies: Mailing, Reminders, and FIT Decision

Will your organization roll-out the program for all clinical practices, a sub-set of practices, or use an opt-in to the program model?

Does the organization want to mail an introduction letter before the FIT to raise awareness? Or conduct other pre-FIT outreach, such as texts or phone calls?

Is your organization using one FIT for all clinical practices?

If not, do the participating clinical practices have an acceptable, high quality FIT?

To run a mailed FIT program, you want a high quality, easy to use FIT test for your population to complete.

Will you have clinical practices validate or scrub your organization's list of eligible patients? Are you able to divide/generate an eligible patient list by clinical practice?

- When patient lists are scrubbed, they can be more accurate in terms of eligible population. However, this process can be time consuming when done by clinic staff.

**Implementation Strategies: Mailing, Reminders, and FIT Decision** (continued)	
Will you use a vendor to mail out the introduction letter?<br>How will you notify clinics when the introduction letter has been mailed?	
Do you want to remove letters returned with bad addresses from the FIT mailing?	
Do you want to include a CRC Fact Sheet and Wordless instructions in the mailing?	
Will your organization use a central FIT return address (such as a lab vendor)? Or use the clinical practices or labs as the return address for FITs?<br>• If a health plan, for example, wants to use the FIT typically used by clinical practices participating in outreach, the returns will be distributed.	
• How will you pay for return postage? Can you establish a business reply USPS code? (Note: a business reply code means you are only billed postage for returned FIT tests, and do not have to stamp all the outgoing reply envelopes.)	
Will your organization do a rolling mailing or a once-a-year mailed approach?	

Patient / Health Plan Member Reminders

Will your program use reminder phone calls after the FITs are mailed?

Who will make phone calls to remind patients about screening? Will calls be made by the lead organization (e.g., Health Plan) or in the clinical practices?

- Clinical practices may have a closer relationship to the population, but less staffing capacity to make phone calls.

Will there be a text reminder from either the health plan or clinical practices?

Incentives

Does your organization want to offer incentives for FIT completion? (NOTE: Evidence shows that incentives can be helpful for first-time responses and certain populations, but not necessarily long-term encouragement.)

Next Steps for Readiness to Implement

- Determine Organizational team: Project Lead, Vendors, Data Specialist
- Determine FIT mailing plan: one time or multiple mailings and frequency
- Determine whether calls or reminders in another format will be made and by whom (vendor, lead organization, or clinical practices)
- Gather current CRC screening data for evaluation of program

Choose your Fecal Immunochemical Test (FIT)

A high-quality, effective, easy to use FIT is critical to any program involving mailed FIT. Before you start a program, you need to determine which FIT or selection of FITs you will use in your program. FITs could be a single central FIT purchased by your organization, supplied by a lab or vendor that will process returned FITs, or supplied by the clinical practices taking part in the program.

For a detailed breakdown on the best options for FIT tests, see:

Clinician's Reference: Stool-Based Tests For Colorectal Cancer Screening
https://nccrt.org/resource/fobt-clinicians-reference-resource/

It explains the different types of stool-based tests available and includes information on sensitivity and specificity for many commonly used tests.

Working with a Vendor for Implementation

Using a print and mailing vendor can take the burden of prepping FITs, printing instructions, and stuffing envelopes away from your organization's staff. Many vendors exist that can prep and mail FIT tests on your behalf. Different vendors have the capacity to implement various components of a CRC screening outreach program, and pricing for these services can vary quite dramatically from one vendor to another. For example, some vendors are equipped to receive, process, and produce results for completed FITs. Other vendors can make reminder phone calls to your population to encourage them to complete and return their FITs. Using a print mailing vendor can help to eliminate confusion and administrative burden, so your clinic can focus on conveying the correct results to your patients. It can lead to improved communication with your patient population and more timely feedback.

How to Choose a Vendor

If your organization decides to use a vendor to implement a mailing outreach program, your organization can follow the guideline below. Mailings can be divided by clinical practices or can be structured to go out on a rolling basis, such as monthly or quarterly.

In a mailed outreach program using FIT tests, the Health Plan provides several items to the print vendor:

Phase One	Phase Two
• A patient list (names and addresses) for all eligible patients, divided by clinical practice name (if applicable)<br>• The print-ready Introduction Letter<br>• Return addresses for envelopes	• The print-ready FIT Insert Letter<br>• The print-ready Fact Sheets, one-page graphical FIT instructions (if applicable)<br>• FIT to be mailed and packaging that comes with FIT mailing<br>• Return addresses and outbound and reply postage instructions

Some disadvantages may include confusion amongst patients if they do not recognize who the test is coming from. If you have not communicated with your patients about a FIT mailing initiative, this may lead to tests being thrown out. Distrust amongst certain populations such as Native American tribes may also be of concern, as they may not want their personal healthcare information shared with a third party. If communication with this vendor is not structured regularly, there may also be difficulty in receiving timely results back.

Establishing a Learning Collaborative

To bring together all the partners implementing a CRC screening outreach, we recommend establishing a regularly occurring meeting between the clinical practices who will be part of the outreach and the lead organization who will be handling the logistics. These meetings can build rapport between parties, give an opportunity to provide a program overview prior to beginning, give updates as outreach activities are ongoing, and answer any questions. We have included in the resources a cadence of agendas and topics to match a mailed FIT program roll-out combined with patient navigation to follow-up colonoscopy.

Potential Attendees: Leads from the participating organizations (e.g., clinical practice managers, QI Population leads, nursing or MA supervisors) and over time addition staff such as patient navigators

Objectives:

- Introductions, engagement of implementation partners
- Provide overview of the project and the population being engaged
- Share ongoing implementation activities (eligible patient list review, mailing procedures, FIT returns, etc.)
- Presentation of evidence-based examples either from participating practices or guest speakers
- Share out of progress and questions from participating organizations
- Expert presentations on CRC screening from content experts
- Sharing new or modified educational and promotional materials, such as flyers or clinic posters
- Discuss next steps in the outreach

As the front-line staff doing this work, the practice is likely acutely aware of the barriers for colorectal cancer screening. The first meeting will be an opportunity to verify what they already know; highlight the disparities in historically underserved populations, including rural and frontier areas, and Medicaid patients; and provide a high-level overview that mailed outreach and patient navigation programs work to reduce these barriers and disparities. Later meetings will share the numbers of eligible patients, any tools for scrubbing and determining the eligible population, mailing progress, FIT return numbers across organizations, and navigation activities.

After the Meeting

- Write up meeting notes and circulate it to all parties invited to attend the meeting series
- Follow up with the participating organizations via email to thank them for their time and participation and include a PDF version of the slide deck

Citations

1. Community Preventive Services Task Force (CPSTF). *The Community Guide.* https://www.thecommunityguide.org/findings/cancer-screening-multicomponent-interventions-colorectal-cancer.html

2 Coury J, Miech EJ, Styer P, Petrik AF, Coates KE, Green BB, Baldwin LM, Shapiro JA, Coronado GD. What's the "secret sauce"? How implementation variation affects the success of colorectal cancer screening outreach. Implement Sci Commun. 2021 Jan 11;2(1):5. doi: 10.1186/s43058-020-00104-7. PMID: 33431063; PMCID: PMC7802298.
