# Supplementary material for: Scale-up evaluation results of a multi-level approach using mailed immunochemical test (FIT) and patient navigation to increase colorectal cancer screening rates in rural clinic settings
Source: BMC Prim Care. 2026 Jul 14;27:361. doi: 10.1186/s12875-026-03428-w (PMC13656510; doi:10.1186/s12875-026-03428-w)
Supplement: Supplementary file 2 [file 12875_2026_3428_MOESM1_ESM.docx]

| **ECHO Session Topics** | |
| --- | --- |
| **Session** | **Didactic Topic** |
| 1 | What is the Business Case for Colorectal Cancer Screening Outreach? |
| 2 | Ways to Successfully Build Engagement and Implement a Collaborative Cancer Screening Program |
| 3 | Designing a Mailed FIT (Fecal Immunochemical Test) Program that Works for your Organization |
| 4 | Identifying your Patients due for Colorectal Cancer Screening |
| 5 | Patient Outreach and Communication: Kit Mailing, Reminders and Follow-Up |
| 6 | Navigating Patients to Follow-up Colonoscopy |
